# Electrically-Conductive Sub-Micron Carbon Particles from Lignin: Elucidation of Nanostructure and Use as Filler in Cellulose Nanopapers

**DOI:** 10.3390/nano8121055

**Published:** 2018-12-15

**Authors:** Janea Köhnke, Harald Rennhofer, Christoph Unterweger, Notburga Gierlinger, Jozef Keckes, Cordt Zollfrank, Orlando J. Rojas, Wolfgang Gindl-Altmutter

**Affiliations:** 1 Department of Materials Science and Process Engineering, BOKU-University of Natural Resources and Life Science, Vienna, 3430 Tulln, Austria; janea.koehnke@boku.ac.at (J.K.); harald.rennhofer@boku.ac.at (H.R.); 2 Wood K plus—Kompetenzzentrum Holz GmbH, 4040 Linz, Austria; c.unterweger@wood-kplus.at; 3 Department of Nanobiotechnology, BOKU-University of Natural Resources and Life Science, Vienna, 1190 Vienna, Austria; notburga.gierlinger@boku.ac.at; 4 Department of Materials Physics, Montanuniversität of Leoben, 8700 Leoben, Austria; keckes@unileoben.ac.at; 5 Chair for Biogenic Polymers, Technische Universität München, 94315 Straubing, Germany; cordt.zollfrank@tum.de; 6 Department of Bioproducts and Biosystems, School of Chemical Engineering, Aalto University, 00076 Aalto, Finland; orlando.rojas@aalto.fi

**Keywords:** carbon particles, cellulose nanopaper, electrical conductivity, lignin

## Abstract

Carbon particles were produced from kraft lignin through carbonization of perfectly spherical, sub-micron beads obtained by aerosol flow. The structure of the resulting carbon particles was elucidated and compared to that derived from commercially available technical lignin powder, which is undefined in geometry. In addition to the smaller diameters of the lignin beads (<1 µm) compared to those of the lignin powder (100 µm), the former displayed a slightly higher structural order as revealed by X-ray diffraction and Raman spectroscopy. With regard to potential application in composite structures, the sub-micron carbon beads were clearly advantageous as a filler of cellulose nanopapers, which displayed better mechanical performance but with limited electrical conductivity. Compression sensing was achieved for this nanocomposite system.

## 1. Introduction

Lignin is the second-most abundant plant bioresource after cellulose. Technical lignin arises as a by-product of the pulping process and is predominantly used for the production of energy. It has been shown that the pulp and paper sector may significantly benefit from a transition from a singular focus on pulp fiber production towards a broader utilization of lignocellulose in a biorefinery framework [1]. In order to enable this transition, value-added utilization pathways for lignin are urgently needed. In this context, the potential applications discussed are diverse and include production of bioaromatics, biopolymers, and biofuel [2,3,4,5,6,7,8,9,10,11,12,13,14,15,16,17,18,19]. In terms of lignin-derived materials, conversion by means of carbonization is attractive, due to the higher carbon content and yield of lignin, e.g., compared to those from cellulose, and the potentially high value of carbon materials obtained in the process [20]. Lignin-derived carbon fibers have been the subject of various studies [21,22,23,24,25,26,27,28,29], but lack mechanical performance relative to their fossil-derived counterparts. Even so, lignin-derived carbon fibers may be attractive as a low-cost and moderate performance alternative for widespread application in the automotive sector, where lightweight material alternatives to steel are needed to reduce fuel consumption [28]. Besides high mechanical performance, high specific surface area carbon materials, both fibrous and particulate, are highly attractive. A high specific surface area may be obtained by the electrospinning of lignin-containing aqueous solutions [22,25,30,31,32,33,34] or from particulate lignin [35,36,37,38,39], both of which can be carbonized. Potential applications of this class of materials lie predominantly in electrochemistry and filtration [7,20,40,41]. 

Lignin-derived and electrically conductive carbon has been discussed recently [42,43,44]. It has been demonstrated that, compared to fossil-based carbon black, moderate electrical conductivity may be obtained. In turn, when such materials are used as filler, they endow polymeric matrices with electrical conductivity. On the downside, however, lignin-derived carbon particles are expected to impair the mechanical performance of the composite materials, especially affecting their tensile strength [45]. According to composite micromechanics, such degradation in tensile strength upon addition of particulate filler diminishes with decreasing particle size [45]. The particle size of commercial, shape-amorphous lignin is in the order of 10 µm–100 µm [6,36,42,46], whereas the diameter of lignin particles obtained by spray-drying of pulping liquor is 1 µm–5 µm [43]. Several preparation procedures for submicron- to nano-scale lignin particles have been reported [6,9,46,47], and recently a high-throughput technology for submicron- and nanolignin synthesis via an aerosol flow reactor was introduced by Ago et al. [48], which has been shown to be cost-competitive and scalable [49]. Most relevantly, the method allows control of size, wettability, and surface morphology (wrinkled or smooth surfaces) [50]. In the present study, we obtained sub-micron carbon particles from spherical lignin beads according to this latter procedure [48] and compared the structures with commercially available (and amorphous) lignin powder, aiming to identify potential advantages of size diminution and shape toward the manufacture and use of carbon particles.

## 2. Materials and Methods 

### 2.1. Materials

Indulin AT lignin was purchased from Meadwestvaco and used as received for reference purposes. The chemistry of this commercially available technical lignin is describes in detail in Reference [51]. Spherical and sub-micron lignin particles or beads were produced according to Ago et al. [48,49]. Briefly, the procedure involved the generation of small droplets of lignin solution in dimethylformamide via a jet atomizer that used nitrogen gas as the carrier, subsequent drying at 153 °C in a heated laminar flow reactor, and fractionation with a low-pressure impactor.

### 2.2. Carbonization

The two types of lignin powders were thermostabilized by slow heating at a rate of 0.01 °C min^−1^ from ambient temperature up to 250 °C in a Memmert oven. Slow heating in an ambient atmosphere induces oxidative changes in the lignin structure, resulting in the elimination of any thermoplastic behavior [52]. Thus, fusing of lignin particles during the initial heating phase of a carbonization process is prevented. Carbonization was carried out in a GERO HTK8 oven with a volume of 6 liters. An argon atmosphere (150 L h^−1^) was used and heating was performed at rates of 1 °C min^−1^ up to 500 °C (1 h holding step), 5 °C min^−1^ up to 900 °C (1 h holding step), and a final step-up to 2000 °C at a rate of 5 °C min^−1^, concluding with a final holding step of 1 h.

### 2.3. Preparation of Carbon-Filled Cellulose Nanopapers

In order to combine high strength and electrical conductivity, cellulose nanopapers with a carbon content of 15 wt.% were prepared by mixing the respective type of carbon particles with an aqueous cellulose nanofibril (CNF) suspension followed by filtration to remove water. The CNF was prepared from elementary chlorine free-bleached kraft pulp from spruce and pine wood and purchased as an aqueous suspension with a dry content of 5.9 wt.% from the Swiss Federal Laboratories for Materials Science and Technology (EMPA). The suspension was diluted to 1.0 wt.% with deionized water and fibrillated by 100 passes at 700 bar in an APV 1000 high pressure homogenizer (SPX Flow Technology, Charlotte, NC, USA). For the preparation of the carbon/CNF mixtures, 42.5 g of the 1% CNF-suspension (equivalent to 0.425 g of dry CNF) and 0.075 g of oven dry carbon powder were poured into a beaker and filled up to a total of 200 g with deionized water. Relying on the known stabilizing function of nanocellulose on carbon nanomaterials [53], no additional dispersant was added. The resulting 0.25 wt.% suspension was homogenized with an Ultra-Turrax T10 (IKA-Werke, Staufen, Germany) basic disperser operated at 18,000 min^−1^ for 2 min. The suspension was then left to stand for 24 h after which it was again homogenized as described above. In order to optimize the dispersion of carbon particles, the suspension was sonicated using a Q500 Sonicator (QSonica, Newtown, CT, USA) equipped with a 0.5-inch diameter titanium probe. Sonication was carried out at a frequency of 20 kHz in pulse mode (50% output intensity) for a total of 3 min. The suspension was filtrated by means of a 1000 mL DURAN glass filtration apparatus equipped with a hydrophilic PVDF membrane filter (Merck No. DVPP09050) with a pore size of 0.65 µm. After filtration, the still wet CNF cake was carefully removed from the filter disc, sandwiched between two PVDF membrane filters and vacuum-dried at 93 °C and 0.1 bar for around 15 min in a Rapid-Koethen paper sheet former (Frank-PTI, Birkenau, Germany). The resulting carbon/CNF films had a diameter of 78 mm and a thickness of 0.2 ± 0.05 mm.

### 2.4. Characterization

Scanning electron microscopy was performed in a Quanta™ 250 FEG from FEI in high-vacuum secondary electron mode. For non-carbonized lignin specimens and carbon-filled cellulose nanopapers, sputter-coating with a thin gold layer was carried out prior to SEM. The electrical resistivity of pure carbon particle powders was measured at different pressures according to References [54,55,56]. Briefly, a volume of 0.2 cm^−3^ carbon powder as produced in the carbonization oven was placed into a polycarbonate cylinder sealed with a conducting aluminum sheet at its bottom. A steel piston was then inserted into the top end of the cylinder. The electrical conductivity of the powder was determined between the bottom and the top end of the cylinder while simultaneously exerting pressure on the piston. The first conductivity value was taken at a pressure of 0.05 MPa.

Wide angle X-ray diffraction was performed with a Rigaku SmartLab 5-Axis X-ray diffractometer using glass capillaries and Cu K alpha radiation with a wavelength of λ = 0.15418 nm. Intensity data were recorded in intervals of 0.02° between the scattering angles 2θ = 5° and 50°. Small angle X-ray scattering (SAXS) was carried out with a Rigaku S-Max3000 3-pinhole SAXS camera with MM002+ source and a Triton 200 multiwire detector. Scattering images were recorded in the range from *q* = 0.08–8 nm^−1^, with *q* = 4 sinθ/λ the scattering vector, integrated and background corrected before further data evaluation.

Raman spectra were acquired using a Raman microscope (alpha300RA, WITec GmbH, Ulm, Germany) equipped with a green laser (λ = 532 nm), spectrometer with a 600 gmm^−1^ grating (UHTS 300 WITec, Ulm, Germany) and a CCD camera (Andor DU401ABV, Belfast, UK). The laser power was set to 5 mW and with an integration time of 0.05 s single spectra were collected every 250 nm over a region of 20 × 20 µm^2^ (6400 spectra/region) using a 100× oil immersion objective (numerical aperture (NA) = 1.4). Finally, average spectra of three regions of the K and L samples were calculated (19,200 spectra/sample) and compared.

Cellulose nanopapers were characterized by means of tensile testing in a Zwick-Roell 20 kN universal testing machine equipped with a 10 kN load cell. Parallel strips with a width of 8 mm and a free length of 50 mm were strained at a rate of 1 mm min^−1^ until fracture. The suitability of carbon-filled cellulose nanopaper for compression sensing was evaluated by sandwiching nanopapers between steel foils and compressing them along their z-axis while measuring electric resistivity via the steel foil sandwich, which was electrically insulated from the testing machine.

## 3. Results and Discussion

### 3.1. Morphology

**Figure 1:**
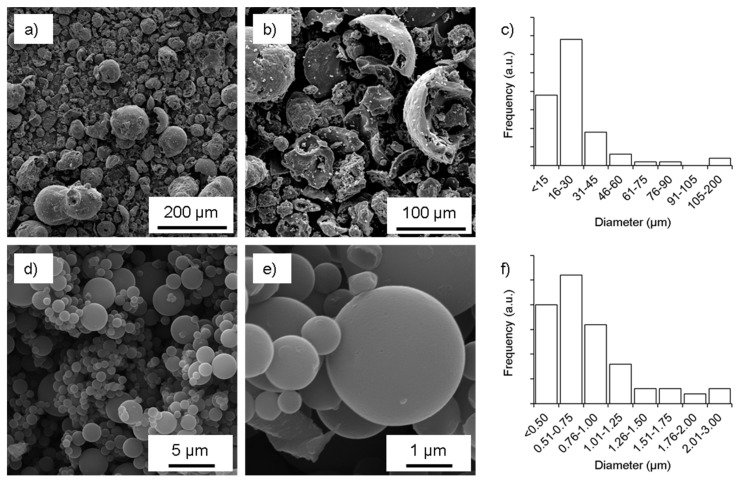
Scanning electron microscopy and diameter distribution (number frequency) of carbon particles derived from Indulin AT (**a**–**c**) and sub-micron lignin particles (**d**–**f**).

The morphology of sub-micron lignin-derived carbon particles is shown in comparison to carbon particles derived directly from as-received Indulin AT (Figure 1). Characteristically, Indulin AT yields geometrically undefined carbon particles with sizes between 10 µm and 100 µm. The surface of these particles was rough and internal cavities were typical. While a small fraction of particles was spherical, most of them presented irregular shapes. In comparison, sub-micron particles with characteristic size in the order of 50 nm to several 100 nm were nearly perfectly spherical in shape and smooth on the surface (note that wrinkle particles can be obtained with the same setup but were not considered in this investigation [50]). No signs of cavities were observed for these particles and it can be assumed that they were solid.

### 3.2. Structural Features

**Figure 2:**
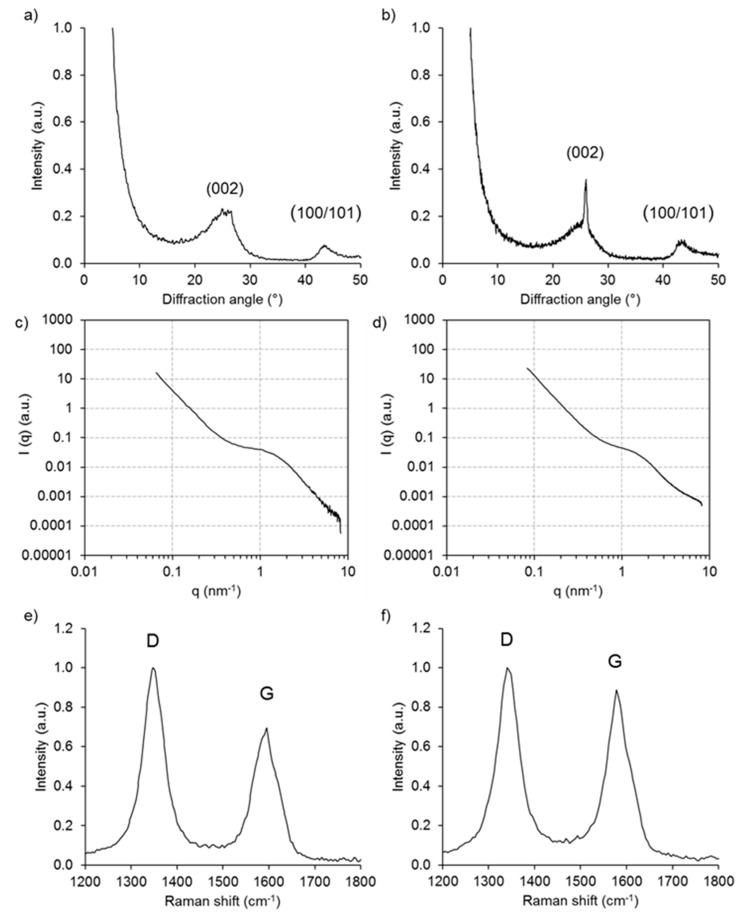
X-ray diffraction (**a**,**b**), small angle X-ray scattering (**c**,**d**), and Raman spectroscopy (**e**,**f**) of carbon particles derived from Indulin AT (**a**,**c**,**e**) and sub-micron lignin particles (**b**,**d**,**f**).

The results of extensive structural characterization of the two types of lignin-derived carbon particles are shown in Figure 2. X-ray diffraction (Figure 2a,b) reveals the presence of graphitic domains in both particle types, as indicated by the characteristic 002 diffraction peak originating from the stacking of graphene sheets in graphite [57]. When comparing the two carbon particles, this peak was more intense for the sub-micron spherical lignin-derived carbon. However, when compared to carbon derived directly from spray dried kraft black liquor in a different study [43], the ordered graphitic character was rather low. In the latter study, unpurified process liquors were carbonized. Small-angle X-ray scattering provides information about the internal pore structure of carbon materials. No significant differences were found in the scattering intensity distribution of the two types of carbon particles tested (Figure 2c,d). The pore radius was 1.09 ± 0.06 nm for Indulin AT carbon and 1.02 ± 0.04 for sub-micron lignin carbon. The corresponding surface area calculated from the scattering curves was 950 ± 140 m^2^ cm^−3^ for Indulin AT and 860 ± 100 m^2^ cm^−3^ for sub-micron lignin carbon.

The finding of only moderately ordered graphitic structure for the carbons produced in the present study was corroborated by Raman spectroscopy (Figure 2e,f). Here, the G-band at ∼1580 cm^−1^, which is associated with a highly ordered graphitic lattice, and the D-band at 1350 cm^−1^, which represents signal from a disordered graphitic lattice [58], were evaluated. While in highly ordered graphite the G band is usually much more intense than the D band, this was not the case for the materials studied here. On the contrary, for both carbon particles the D band was the most intense, indicating only a low content of ordered graphitic domains. Using the ratio between the peak intensities of the D and G peak as an indicator, *I_D_*/*I_G_* was 1.13 for Indulin-derived carbon and 1.45 for sub-micron lignin derived carbon. This is similar in magnitude compared to *I_D_*/*I_G_* values of 1.25–1.26 obtained for mesoporous carbon obtained from lignin nanofiber networks [59], but significantly higher than *I_D_*/*I_G_* of 0.25 and 0.60 obtained for carbon produced by direct carbonization of kraft and sulphite pulping liquors, respectively. Nevertheless, both X-ray diffraction and Raman spectroscopy of the sub-micron lignin-derived carbon showed a tendency towards higher graphitic order compared to carbon prepared directly from Indulin AT powder. 

### 3.3. Electrical Conductivity

**Figure 3:**
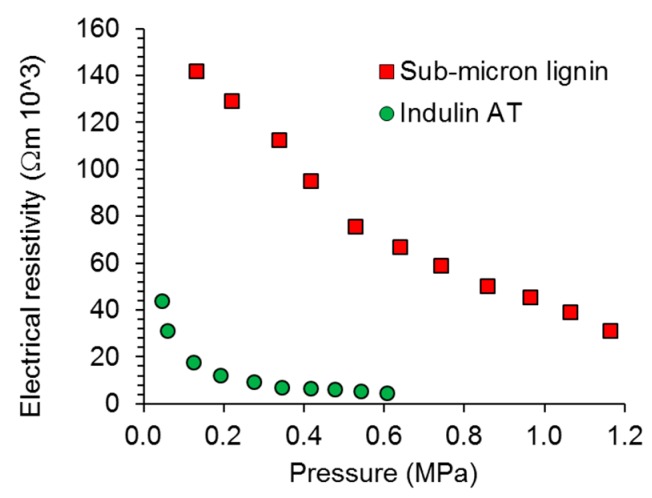
Pressure-dependent electrical resistivity of the two types of lignin-derived carbon powder.

The electrical conductivity of the obtained carbon materials is of primary interest within the present study. As shown in Figure 3, sub-micron lignin carbon showed significantly less conductivity than that derived from Indulin AT. While the latter showed conductivity in a range comparable to earlier results with lignin-derived carbon [42,43,44], sub-micron lignin carbon clearly showed poor performance. In this context, however, it has to be considered that the density of both carbon powders was significantly different during this experiment. During the conductivity measurement, the density of Indulin AT derived carbon was between 0.50 g cm^−3^ and 0.51 g cm^−3^, whereby density increased linearly as increasing pressure was applied. For the sub-micron lignin derived carbon, the density was only between 0.28 g cm^−3^ and 0.29 g cm^−3^. Thus, on the basis of mass, the conductivity difference between both powders was still present, but was less pronounced.

### 3.4. Carbon-Filled Cellulose Nanopapers

Electrically conducting carbon may be used to endow a polymeric matrix with strain-sensing capability [44]. For this purpose, the matrix is filled with conducting filler at a content above the electric percolation threshold [60]. Tensile strain is then applied, which leads to an increasing distance between conductive filler particles and hence decreased electrical conductivity. In order not to impair the mechanical performance of the polymeric matrix, a small filling ratio is usually desired. In view of the relatively poor electrical conductivity of lignin-derived carbon obtained in the present study, this approach was deemed unsuitable because a high filling ratio of >20% would be required, as inferred from the percolation threshold of 20% carbon content determined in an earlier paper [44].

**Figure 4:**
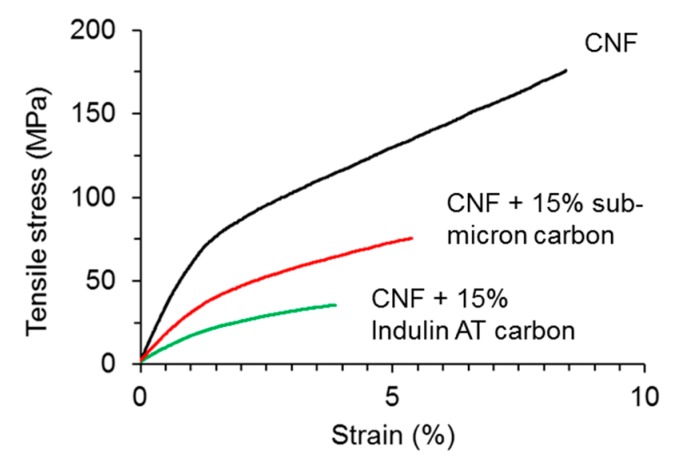
Representative stress-strain curves from tensile tests with pure cellulose nanopaper (CNF) and nanopaper filled with 15 wt.% carbon particles.

Instead, we evaluated the potential of the carbon particles as fillers in a setup used to assess the suitability of bio-based carbon in the manufacture of cellulose nanopaper for compression sensing. As shown in Figure 4, the presence of 15 wt.% carbon particles in CNF nanopaper significantly diminished the mechanical performance of this material. While pure CNF nanopaper showed a tensile modulus of 6.8 ± 0.32 GPa and strength of 195 ± 19 MPa, these values were reduced to 3.0 ± 0.08 GPa and 75 ± 3 MPa for sub-micron carbon, and 1.7 ± 0.01 GPa and 36 ± 1 MPa for Indulin AT carbon, respectively.

**Figure 5:**
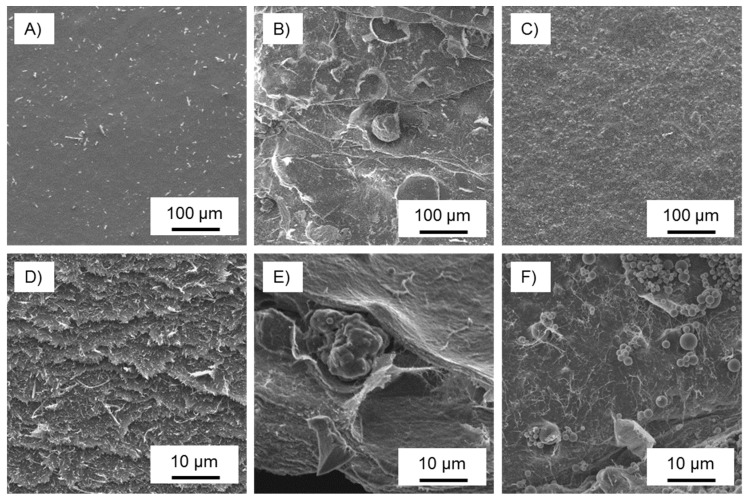
Scanning electron microscopy of the surface of pure cellulose nanopaper (**A**), nanopaper filled with 15 wt.% Indulin AT carbon (**B**), nanopaper filled with 15 wt.% sub-micron carbon (**C**), and corresponding fracture surfaces (**D**–**F**).

The results were as expected; namely, the carbon filler diminished the mechanical properties of cellulose nanopapers since there was a clear lack of interfacial adhesion between the carbon and the cellulose fibrils. Even so, nano-scale lignin may be beneficial to cellulose nanopaper mechanics, as it enhances the colloidal stability and dispersity of CNF in aqueous dispersions, and suppresses fibril aggregation during film formation [61]. What was surprising was the remarkably better performance of the sub-micron carbon over Indulin AT carbon. Clearly, the reduced particle size of the carbon filler, to the sub-micron size range, is an advantage [45]. Inquiring more deeply into the reasons for these observations, scanning electron microscopy (Figure 5) revealed a smooth surface for pure cellulose nanopaper. In contrast, the surface of nanopaper filled with Indulin AT carbon appeared rough, with several individual carbon particles with a diameter of 70–100 µm clearly visible. Sub-micron carbon filled nanopaper also appeared more structured than the smooth surface of pure cellulose nanopaper. However, the small sub-micron carbon particles appeared quite homogeneously distributed at this level of magnification. Images of fracture surfaces taken after tensile testing confirmed severe structural disorder due to the presence of comparably large carbon particles in Indulin AT filled nanopaper. By comparison, the structure of sub-micron lignin filled nanopaper appears much less disturbed due to the small size of carbon particles of this variant.

**Figure 6:**
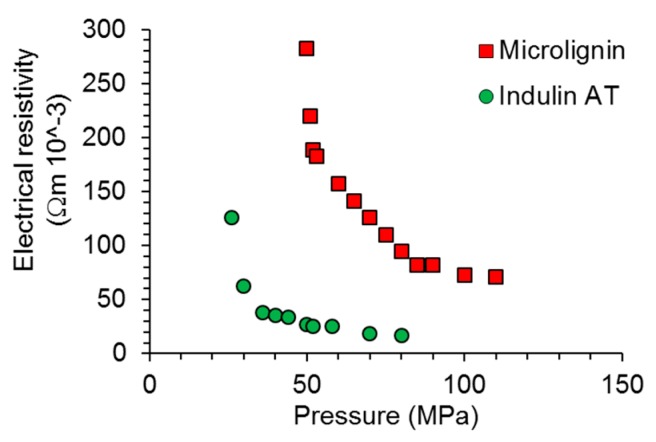
Pressure-dependent electrical resistivity along the out-of-plane direction of cellulose nanopapers filled with the two types of lignin-derived carbon particles.

Most remarkably, compared to the values of electrical resistivity determined for the neat carbon powders, the resistivity of carbon-filled cellulose nanopapers was orders of magnitude higher. The nanopapers filled with 15 wt.% carbon showed electrically insulating behavior at the beginning of a compression experiment (Figure 6); electrical current was registered starting from 25 MPa pressure for Indulin AT carbon and from 50 MPa onwards for nanopapers filled with sub-micron lignin carbon. Thus, in principle, composite material configurations such as the carbon-filled nanopaper might serve as compression sensors with moderate sensitivity at high loads, but their practical usefulness has yet to be determined.

## 4. Conclusions

The results presented above show that carbon particles with partially ordered graphitic structure and limited electrical conductivity can be produced from sub-micron lignin particles. Overall, sub-micron lignin derived carbon did not show significant differences in terms of structure and electrical conductivity compared to carbon produced from as-received Indulin AT lignin, with particle sizes ranging from 10 µm to 100 µm. The only very clear advantage of sub-micron lignin-derived carbon particles compared to their larger counterparts lies in their positive effect on tensile behavior. At a filler content of 15 wt.% sub-micron carbon filled cellulose nanopapers performed twice as well in terms of tensile strength and stiffness compared to nanopaper filled with Indulin AT-derived carbon.
